# Seven-year kinetics of RTS, S/AS01-induced anti-CSP antibodies in young Kenyan children

**DOI:** 10.1186/s12936-021-03961-2

**Published:** 2021-12-02

**Authors:** Robert M. Mugo, Kennedy Mwai, Jedidah Mwacharo, Faiz M. Shee, Jennifer N. Musyoki, Juliana Wambua, Edward Otieno, Philip Bejon, Francis M. Ndungu

**Affiliations:** 1 KEMRI-Wellcome Trust Collaborative Programme, P.O. Box 230, Kilifi, 80108 Kenya; 2 Institute of Immunology, Center for Infection Medicine, Freie Universtät Berlin, 14163 Berlin, Germany; 3 Department of Biological Sciences, Pwani University, P.O. Box 195-80108, Kilifi, Kenya; 4 Centre for Tropical Medicine and Global Health, Nuffield Department of Medicine, University of Oxford, Oxford, UK; 5 Division of Infectious Diseases, Department of Medicine Solna and Centre for Molecular Medicine, Karolinska Institute, Stockholm, Sweden

**Keywords:** RTS,S/AS01, Vaccines, Antibodies, *Plasmodium falciparum*, Circumsporozoite protein

## Abstract

**Background:**

RTS,S/AS01, the leading malaria vaccine has been recommended by the WHO for widespread immunization of children at risk. RTS,S/AS01-induced anti-CSP IgG antibodies are associated with the vaccine efficacy. Here, the long-term kinetics of RTS,S/AS01-induced antibodies was investigated.

**Methods:**

150 participants were randomly selected from the 447 children who participated in the RTS,S/AS01 phase IIb clinical trial in 2007 from Kilifi-Kenya. Cumulatively, the retrospective follow-up period was 93 months with annual plasma samples collection. The levels of anti-CSP IgM, total IgG, IgG1, IgG2, IgG3, and IgG4 antibodies were then determined using an enzyme-linked immunosorbent assay.

**Results:**

RTS,S/AS01 induced high levels of anti-CSP IgG antibodies which exhibited a rapid waning over 6.5 months post-vaccination, followed by a slower decay over the subsequent years. RTS,S/AS01-induced anti-CSP IgG antibodies remained elevated above the control group levels throughout the 7 years follow-up period. The anti-CSP IgG antibodies were mostly IgG1, IgG3, IgG2, and to a lesser extent IgG4. IgG2 predominated in later timepoints. RTS,S/AS01 also induced high levels of anti-CSP IgM antibodies which increased above the control group levels by month 3. The controls exhibited increasing levels of the anti-CSP IgM antibodies which caught up with the RTS,S/AS01 vaccinees levels by month 21. In contrast, there were no measurable anti-CSP IgG antibodies among the controls.

**Conclusion:**

RTS,S/AS01-induced anti-CSP IgG antibodies kinetics are consistent with long-lived but waning vaccine efficacy. Natural exposure induces anti-CSP IgM antibodies in children, which increases with age, but does not induce substantial levels of anti-CSP IgG antibodies.

**Supplementary Information:**

The online version contains supplementary material available at 10.1186/s12936-021-03961-2.

## Background

Malaria is a life-threatening disease, causing approximately 229 million cases and about 400,000 deaths in 2019 worldwide [1]. Wide-scale malaria control efforts have been implemented in malaria-endemic countries, including indoor residual spraying of insecticide, sleeping under long-lasting insecticide-treated bed nets, using rapid diagnostic tests for swift diagnosis, and the use of artemisinin-based combination therapy (ACT) for malaria treatment. These efforts have gradually reduced malaria transmission especially for the period between the years 2010–2014 [1]. However, the malaria incidence rate per 1000 population at risk has stalled for the period 2015–2019 [1]. Furthermore, the emergence of widespread pyrethroid resistance coupled with the emerging parasite resistance to ACT in Asia and some parts of West Africa highlights the need for the adoption of novel methods to complement the existing control efforts [2].

RTS,S/AS01 malaria vaccine has been tested in a phase III clinical trial and shown to provide partial protection against clinical malaria [3]. RTS,S/AS01 is a recombinant *Plasmodium falciparum* vaccine based on the circumsporozoite protein (CSP) which is the major protein on the surface of the sporozoites. The vaccine construct has 19 copies of the central repeat region (NANP) which contains known immunodominant B-cell epitopes and the C-terminal, which contains T-cell epitopes fused to hepatitis B surface antigen (HBsAg). The two regions are simultaneously co-expressed with un-fused HBsAg in yeast cells. Co-expression with HBsAg enhances the vaccine immunogenicity and stability [4].

RTS,S/AS01 phase III clinical trial was conducted in seven African countries in thousands of children aged between 5 and 17 months. The children received three vaccine doses at an interval of 1 month and a fourth booster dose after 20 months, with the main endpoints being the occurrence of malaria over 12 months following the final vaccine dose. The phase III trial results were released in the year 2015, which showed vaccine efficacy of 36.3% with a fourth booster dose [3]. This translates to preventing about four in 10 malaria cases. Even though the vaccine efficacy was way below the recommended 75% efficacy by the World Health Organization (WHO), it was endorsed by the European Medicines Agency (EMA) for use in the Expanded Programme on Immunization (EPI) in 2015 [5].

The WHO recommended further vaccine evaluations in large-scale pilot studies in malaria-endemic areas of Kenya, Ghana, and Malawi, which commenced in 2018. Recently, the results from the pilot studies indicated a strong RTS,S/AS01 vaccine safety profile, good feasibility of the vaccine delivery, and high impact in the real-life childhood vaccination setting [6]. Subsequently, the WHO has recommended its widespread use among children in sub-Saharan Africa and other regions with low to moderate *P. falciparum* transmission. RTS,S/AS01 vaccination is expected to reinvigorate the fight against malaria in children.

Though the vaccine-induced antibodies (Abs) wane relatively quickly after primary vaccination, high levels of anti-CSP IgG Abs have been associated with protection from malaria episodes [7, 8]. The maintenance over time of the RTS,S/AS01-induced Abs remains unclear. Some of the blood-stage Abs (MSP1, AMA1, and EBA175) were significantly reduced in the RTS,S/AS01 group 7 years post-vaccination as compared to the control group [9]. Though these specific blood-stage Abs are not consistently associated with immunity to malaria, these findings suggest that RTS,S/AS01 might cause a delayed acquisition of blood-stage natural immunity. This may reverse the vaccine gains by making the vaccinees susceptible to malaria infections as the vaccine-induced immunity wanes later. As such, it is crucial to understand the long-term kinetics and maintenance of these RTS,S/AS01-induced anti-CSP Abs.

This study used stored samples from a longitudinal cohort of children from Kilifi-Kenya within a phase IIb RTS,S/AS01 trial that was also on active weekly surveillance of malaria for the length of this analysis. The levels of RTS,S/AS01-induced anti-CSP total IgG, IgG subclasses, and IgM Abs were measured for both the intervention and control groups longitudinally.

## Methods

### Study design

Four hundred and forty-seven children aged 5–17 months from Junju in Kilifi Kenya which is a malaria-endemic area participated in a randomized, controlled, and double-blind RTS,S/AS01 Phase IIb clinical trial in 2007 [10]. Both the vaccine (RTS,S/AS01) and the control group (rabies vaccine) were eligible for this cumulative 93 months (~ 7 years) follow-up study to help understand the kinetics of vaccine-induced immunity in children exposed to endemic *P. falciparum* transmission.

### Plasma samples

**Fig. 1:**
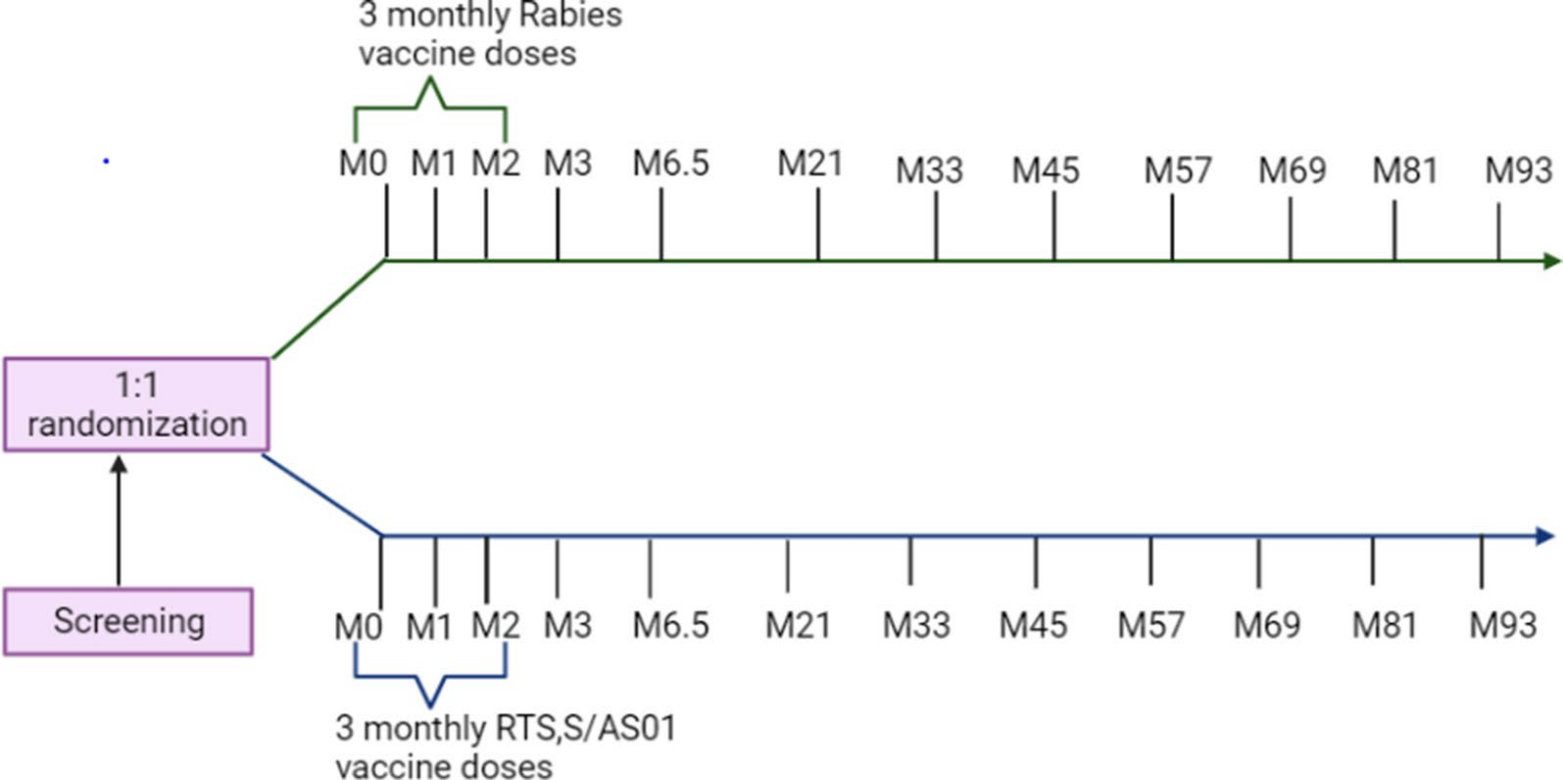
Study design showing the initial RTS,S/AS01 phase IIb clinical trial participants screening, randomization, and the longitudinal plasma samples collection from baseline month (M) M0. This was followed by the administration of 3 monthly doses of either RTS,S/AS01, or rabies vaccines at M0, M1, and M2. The second sample was collected one month after the final dose (M3) whilst the third sample was collected at M 6.5. The subsequent samples were collected annually up to M93

Following randomization into either group, the participants received three monthly doses of either vaccine. Baseline plasma samples for both the vaccine and control groups were collected between January and March 2007, i.e., before receiving the three-monthly vaccine doses of either the RTS,S/AS01, or the rabies vaccine. The second sample was collected one month following the third vaccine dose (June–July 2007). This was followed by annual plasma samples collection between March and May 2008, 2009, and 2010. Further annual plasma samples were collected between March and April 2011, 2012, 2013, 2014, and 2015 (Fig. 1). These sampling timepoints correspond with the malaria peak seasons in the region. These samples have been stored at − 80ºC.

### Sample selection

From the 447 participants who participated in the initial randomization in 2007, 75 participants were randomly selected (using RAND function in Microsoft excel 2019) for each group with at least one plasma sample for each of the 10 timepoints. To determine the levels of the RTS,S/AS01-induced anti-CSP IgG and IgM Abs, plasma samples (n = 75) from all the timepoints, and (n = 50) for the IgG subclasses were tested, respectively. These timepoints were also used for determining the levels and kinetics of anti-tetanus (non-malarial control antigen) IgG Abs responses (n = 50 for each group).

### Experimenter blinding

The experimenters were blinded from the vaccine and the control groups as well as the ages of the participants until all the measurements for anti-CSP IgG, IgM, and anti-tetanus IgG Abs levels were completed.

### Determining the levels of anti-CSP IgG and IgM antibodies

The plasma samples were tested using a standard Enzyme-Linked Immunosorbent Assay (ELISA) for the levels of IgG and IgM Abs against the *P. falciparum* CSP antigen (NANP)9 (Biomatik™). Briefly, high absorbance ninety-six-well plates (Immuno4 HBX-Thermo Scientific™) were coated with 100 µl per well of 1 µg/ml of (NANP)9 antigen in carbonate-bicarbonate coating buffer, pH 9.4 (Sigma Aldrich™) and incubated at 4 °C overnight. The following morning the plates were washed three times with 0.05% Tween 20 in phosphate-buffered saline (PBS-T; pH 7.2) using an ELx405 automated machine washer. Then blocking was done for 3 h for IgG and 6 h for IgM at room temperature (RT) by adding 200 µl per well of 1% dried skimmed milk powder in PBS-T.

Following three washes, 100 µl per well of diluted plasma samples was added to the duplicate wells at a final dilution of 1/4000 for IgG and 1/500 for the IgM in PBS-T and incubated at 4 °C overnight. On the next day, the plates were washed six times with PBS-T, and pat dried. This was followed by the addition of either 100 µl per well horseradish peroxidase-conjugated rabbit anti-human IgG (DAKO™) diluted at 1/5000 in PBS-T for IgG detection or rabbit anti-human IgM (The Binding Site™) at the same dilution for the IgM detection. Then the plates were incubated for 3 h at RT.

The plates were then washed six times with PBS-T followed by pat drying. Then 100 µl per well of OPD (Sigma fast tablets- Sigma #P9187) fast substrate solution was added and the plates were left at RT in the dark for 15 and 30 min for IgG, and IgM Abs, respectively. The reaction was stopped by adding 25 µl per well of 2 M H_2_SO_4_. The optical densities (ODs) were then read at an absorbance of 492 nm using the Gen5™ microplate reader.

### Determining the levels of anti-CSP IgG subclasses

Following antigen coating and blocking as described in the anti-CSP IgG ELISA, 100 µl per well of plasma sample was added to the duplicate wells at a final dilution of 1/500 and incubated overnight at 4 °C. The subsequent steps were as described for IgG/IgM measurements except for the secondary Abs; either sheep anti-human IgG1, IgG2, IgG3, and IgG4 conjugated to HRP (all from The Binding Site™) diluted at 1/5000 in PBS/T was added, respectively.

### Determining the levels of anti-tetanus IgG antibodies

To determine the levels of anti-tetanus IgG responses, a similar ELISA protocol as that of the anti-CSP IgG and IgM Abs was used. However, the plasma sample dilution was 1:1000 and the ODs were read after 15 min of incubation with OPD in the dark at an absorbance of 492 nm. To determine anti-tetanus IgG Abs concentrations for each plate, a standard with a known concentration of 125 international units was used. A twofold dilution was used for the standard curves, starting with 1:2000 dilution.

### Experimental controls and standardization

To ensure accuracy of the measurements, the plasma samples were tested in duplicate. Participants whose samples exhibited high variability i.e., > 20% were re-tested. Besides, six malaria-naïve negative controls from Sweden and the UK were included per plate. The anti-tetanus IgG responses were tested as a non-malaria control antigen for both groups for the entire follow-up period. To minimize within-person variations due to inter-plate variations all the ten timepoints samples for each participant were measured on the same plate.

### Determining arbitrary ELISA units for the anti-CSP Abs

Pooled plasma from 10 participants with the highest antibody titers at M3 was used to establish the standard curves for IgG, IgM, and the IgG subclasses. A twofold dilution was used for the standard curves, starting with 1:2000 for IgG, and 1:250 for both IgM and IgG subclasses. The highest concentration was assigned an arbitrary ELISA value of 100. Subsequently, through subtraction of the blank wells average ODs to correct for the background reactivity and multiplication with the dilution factor, the arbitrary ELISA units (AEUs) for each plate were independently determined using Gene5™ analytical software.

### Statistical analysis

The Mann–Whitney *U* test, which is a non-parametric test was used to determine if the antibody levels changed significantly throughout the follow-up period both within and between the groups for each of the 10 timepoints. *P*-value < 0.05 was considered significant. Linear, non-linear, and mixed-effect modelling were used for further analysis of the Abs kinetics. GraphPad Prism version 8.0 was used to generate the Abs trends whilst R statistical software version 4.1.0 (R Core Team 2019) was used for modelling.

## Results

### Kinetics of RTS,S/AS01-induced anti-CSP IgG, and IgM responses

**Fig. 2:**
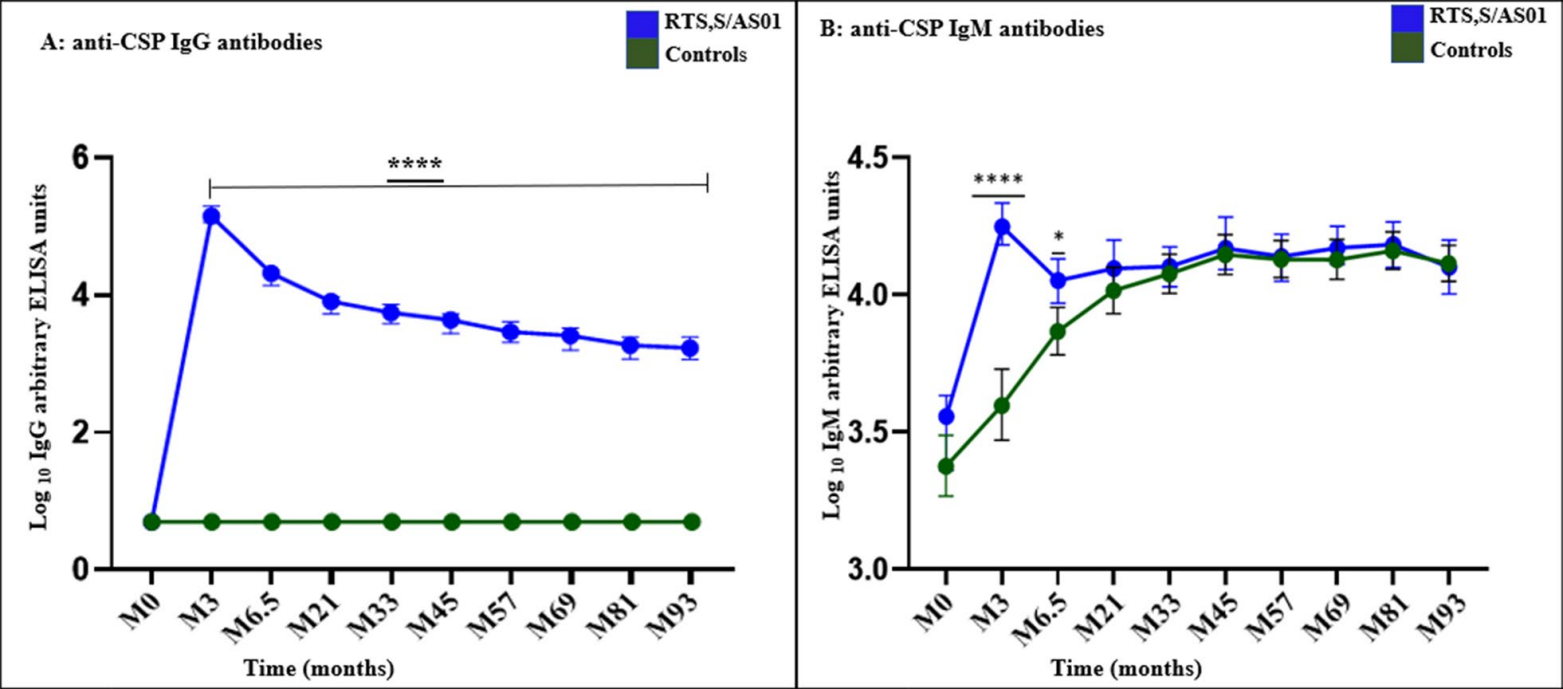
Induction and decay of RTS,S/AS01 anti-CSP IgG (**a**) and IgM (**b**) Abs. The trends were generated using medians. Significance codes: ****P < 0.0001,*P < 0.01; the error bars represent 95% confidence intervals*.* Blue represents the RTS,S/AS01 group, n = 75, green represents the control group, n = 74

Plasma samples collected during the 7 years follow-up period were tested for the IgG and IgM Abs levels in both the vaccine and control groups against the immunodominant central repeat region of *P. falciparum* CSP (NANP)9. The baseline reactivity was minimal for both groups and the anti-CSP IgG and IgM responses exhibited no statistically significant differences between the groups. This was followed by a peak vaccine response one month after the final vaccine dose (M3). The IgG antibodies remained significantly higher in the vaccine group for the 7 years follow-up period (Fig. 2a). As earlier reported in the four years follow-up, most of the control group participants had undetectable levels of anti-CSP IgG Abs for the entire seven years of follow-up [11].

Subsequently, no further analysis is shown on the control group's anti-CSP IgG responses. Contrary, the anti-CSP IgM antibodies remained significantly higher in the vaccine group compared to the controls up to 6.5 months post-vaccination. The control group's anti-CSP IgM levels rose steadily and caught up with the vaccine group by month 21 post-vaccination (Fig. 2b).

Linear and non-linear modelling was used for additional investigation of the anti-CSP Abs decay patterns and boosting. The linear model for RTS,S/AS01 induced anti-CSP IgG response shows that the Abs are gradually heading towards extinction. Both models show no boosting of RTS,S/AS01 anti-CSP IgG antibodies in the vaccine group by natural exposure. Contrary, the control group linear and non-linear model shows anti-CSP IgM Abs boosting by natural exposure, which increases with age (Additional file 1).

### Large individual to individual variations in the RTS,S/AS01-induced anti-CSP IgG Abs responses

To further evaluate the dynamics of the antibody response among the vaccinees a linear mixed-effects model (LMM) was fitted. The LMM applied person as the random effect (δ). The person intraclass correlation coefficient (ICC) describes the similarity of the Abs responses among the participants. The ICC for both vaccine-induced anti-CSP IgG and IgM Abs was 28.0% (n = 75). The controls anti-CSP IgM responses exhibited higher variability among the participants relative to the RTS,S/AS01 group (ICC, 23.0%) (n = 74). Age at the time of vaccination, i.e. any age between (5–17 months) did not have a significant effect on the Abs decay patterns (estimate = − 0.02, 95% CI − 0.11–0.06, *P*-value 0.602). Follow-up time (months) for the vaccine-induced Abs had a significant negative association with the anti-CSP IgG Abs decay (estimate = − 0.04, 95% CI − 0.05 to − 0.04, but showed a significant positive association with IgM Abs kinetics, (estimate 0.004, 95% CI 0.002–0.006). Natural exposure induced anti-CSP IgM antibodies (control group) also showed significant positive associations with follow-up time (estimate 0.021, 95% CI 0.010–0.020) *P*-value < 0.001 (Table 1).

**Table 1:** Linear Mixed-effects models (LMM). The LMM shows the intraclass correlation coefficient (ICC), the effects of age at the time of vaccination, and the follow-up time effect on the vaccine-induced anti-CSP IgG responses for 7 years

Predictors	RTS,S/AS01-induced anti-CSP IgG Abs		RTS,S/AS01-induced anti-CSP IgM Abs		Naturally-induced anti-CSP IgM Abs (control group)	
	Coef. (95% CI)	*P*-value	Coef. (95% CI)	*P*-value	Coef. (95% CI)	*P*-value
Time in years (follow-up)	− 0.040 (− 0.051, − 0.043)	< 0.001	0.004 (0.002, 0.006)	< 0.001	0.021 (0.010, 0.020)	< 0.001
Age (5–17 months) at the time of vaccination	− 0.021 (− 0.110, 0.062)	0.602	0.028 (− 0.010, 0.064)	0.143	0.024 (− 0.031, 0.073)	0.47
ICC (δ)	0.28 (3.69)		0.28 (0.68)		0.23 (1.63)	

ICC, intraclass correlation coefficient, δ, random effect variances

### Kinetics of RTS,S/AS01-induced anti-CSP IgG subclasses

**Fig. 3:**
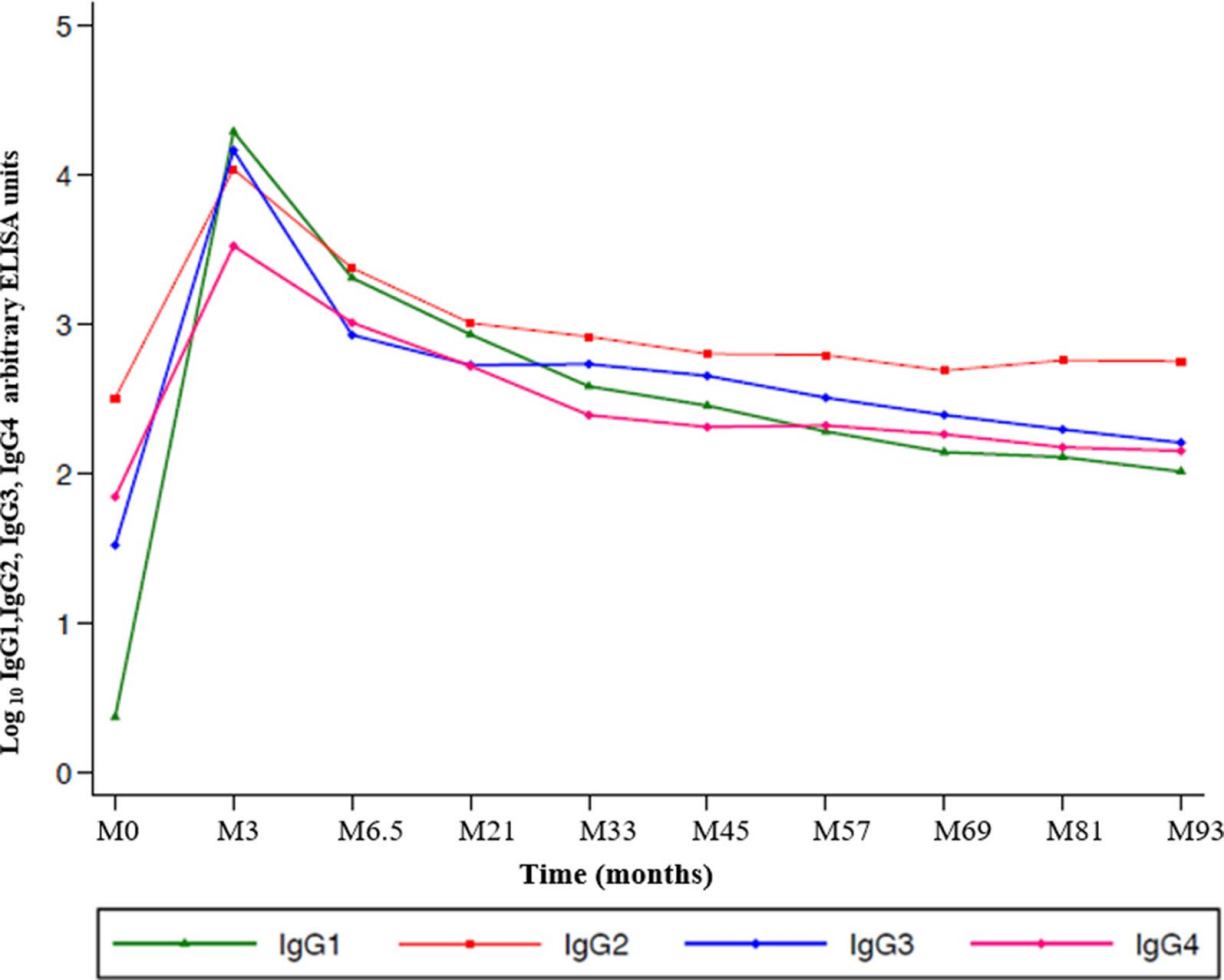
Median trends showing the patterns differences of the RTS,S/AS01-induced anti-CSP IgG subclasses antibodies. Green (IgG1), red (IgG2), blue (IgG3), and pink (IgG4), n = 50 for all the subclasses

The participants were then tested for the RTS,S/AS01 induced anti-CSP IgG subclasses to determine their balance for the entire follow-up period. RTS,S/AS01 induced anti-CSP IgG subclasses were mainly IgG1, IgG3, and IgG2 and to a lesser extent IgG4 at M3. RTS,S/AS01 induced anti-CSP IgG2 Abs predominated the IgG subclasses responses in the later timepoints (Fig. 3).

There were no significant differences in the kinetics of anti-tetanus IgG responses between the RTS,S/AS01, and the control groups for the entire period of follow-up except for M3 (Additional file 2).

## Discussion

Antibodies are key components of both acquired and vaccine-induced immunity to *P. falciparum* infections in humans [12–14]. RTS,S/AS01 induced anti-CSP Abs plays a crucial role in the mediation of vaccine protective immunity [15, 16]. However, most studies on vaccine-induced humoral immunity have largely focused on total anti-CSP IgG Abs responses limited to a maximum of four years follow-up [10, 11, 17]. This study reports that RTS,S/AS01 induces high levels of anti-CSP IgG Abs, which wanes relatively quickly 6.5 months after primary vaccination followed by a slower decay, which is consistent with other studies [10, 11]. The current study shows that the kinetics of the anti-CSP IgG and IgM Abs were different both within and between the vaccine and control groups.

The lack of boosting for the vaccine-induced anti-CSP IgG Abs by the continuous natural exposure might be explained by the slow acquisition of pre-erythrocytic immunity in children [18]. Furthermore, there were no measurable levels of anti-CSP IgG among the controls, suggesting that natural *P. falciparum* infection does not induce substantial quantities of anti-CSP IgG specific memory B cells in children.

Contrary to the RTS,S/AS01 induced anti-CSP IgG Abs responses, the kinetics for the non-malarial control antigen (tetanus) exhibited evident anti-tetanus IgG Abs boosting. As earlier reported, the median anti-tetanus IgG Abs response was slightly higher in the control group at M3 [19, 20]. This might due to random chance with multiple comparisons, or perhaps due to bystander activation of anti-tetanus memory B cells by the rabies vaccination. However, there were no significant differences between the two groups’ anti-tetanus IgG Abs kinetics over the subsequent years.

This study demonstrates that IgM Abs against the NANP region of the CSP was effectively induced following RTS,S/AS01 vaccination. Interestingly, IgM Abs persisted throughout the seven years of the follow-up, suggesting possible induction and subsequent boosting of the IgM memory response upon natural exposure. In support of this view, the control group IgM levels increased steadily due to natural exposure boosting from baseline and caught up with the vaccine group 21 months later*.*

Long-lived IgM responses have also been described in naturally acquired blood-stage immunity to *P. falciparum* in individuals living within malaria-endemic areas [21]. Also, somatically hypermutated memory IgM + B cells were recently reported in humans from regions of natural malaria transmission [22]. In connection with antibodies functionality, the Fulani individuals who exhibit marked natural malaria immunity have been shown to possess higher IgM Abs levels with higher breadth against a wide range of *P. falciparum* antigens [23]. In addition, IgM is more effective in complement activation and fixation than IgG and is associated with a reduced risk of clinical malaria [21, 24].

The fourth RTS,S/AS01 booster dose does not increase the anti-CSP IgM levels contrary to the boosting seen in the anti-CSP IgG Abs responses [25]. Similarly, the minimal boosting of the vaccine-induced anti-CSP IgM Abs seen in this study can be explained by the Abs homeostatic mechanism that prevents overproduction by boosting. In support of this view, the naturally induced IgM Abs levels increased up to the vaccine levels, but not beyond. However, this warrants more research on IgM induction and functionality. Comparably, immunization with two doses of radiated sporozoites led to plateauing of anti-CSP IgM Abs responses in mice [26].

The interactions between RTS,S/AS02 specific Abs with the complement system, specifically via the classical pathway has recently been identified as one of the potential antibody effector mechanisms against the sporozoites. RTS,S/AS02 vaccinated children have been shown to express high levels of C1q-fixing anti-CSP Abs [27]. IgG1 and IgG3 possess the greatest complement-fixing ability, whereas IgG2 and IgG4 exhibit minimal ability [28].

Consistent with other studies, the findings of this study show that RTS,S/AS01 induced anti-CSP IgG subclasses are mainly cytophilic IgG1 and IgG3 followed by IgG2 with IgG4 being the least [29]. Similar findings were reported for RTS,S in the combination with the AS02 adjuvant [27]. Noteworthy, the cytophilic IgG1 and IgG3 waned relatively rapidly following vaccination. IgG2, which is the least functional of the IgG subclasses, predominated in the late timepoints responses*.* These subclasses kinetics suggest that the cytophilic IgG1 and IgG3 are responsible for the early vaccine protective effect but later on, the non-cytophilic IgG2 predominates. The predomination of IgG2 during the later years might have been responsible for the rebound reported five years post-vaccination [30].

The participants in the current study exhibited large variations in the kinetics of the IgG responses. These variations may be caused by genetic factors such as the HLA types or due to the differential amounts of malaria exposure [31, 32]. The variations in individual immune responses (ICC = 28.0%) reported in this study may partly answer the question on the varying vaccine efficacy across the clinical trial sites and the subsequent low vaccine efficacy of about 28.3% in children without the fourth booster vaccination [3]. The linear mixed-effect model showed that time, which may indicate exposure to malaria [11], had minimal but significant negative and positive associations with anti-CSP IgG and IgM Abs decay patterns, respectively. Age at the time of vaccination was not associated with the IgG decay patterns. The lack of significant association between long-term Abs decay and age suggests that any age between 5 and 17 months is effective for the vaccine administration; this is probably because by this age the children’s immune system is relatively well developed.

## Conclusions and recommendations

In summary, this study presents evidence that RTS,S/AS01 vaccination rapidly induces anti-CSP IgG and IgM Abs which persists 7 years post-vaccination. However, the kinetics and maintenance of the anti-CSP IgG Abs differ from that of the anti-CSP IgM Abs. The findings of this study are encouraging regarding the wider use of RTS,S/AS01 considering that there was no booster dose in the Phase IIb trial. Collectively, these findings show the importance of assessing total IgG, IgG subclasses, and IgM in vaccine serological evaluations. More studies should be conducted on the mechanisms of IgM function and protection in both pre-erythrocytic vaccine-induced and natural immunity.

## Supplementary Information

**Additional file 1. **Decay patterns of the anti-CSP IgG and IgM antibodies.

**Additional file 2. **Kinetics of anti-tetanus IgG responses for the RTS,S/AS01, and control groups.

## Data Availability

The datasets used and/or analysed in the current study are available from the corresponding author upon reasonable request.

## Abbreviations

Abs: Antibodies
ACT: Artemisinin-based combination therapy
AEU: Arbitrary Elisa Units
EPI: Expanded programme on immunization
CSP: Circumsporozoite protein
ELISA: Enzyme-linked immunosorbent assay
HRP: Horseradish peroxidase
HBsAg: Hepatitis B surface antigen
ICC: Intraclass correlations coefficient
IgG: Immunoglobulin G
IgM: Immunoglobulin M
M: Month
NANP: Peptide representing the central repeat region of CSP
OD: Optical density
OPD: O-Phenylenediamine
PBS: Phosphate buffered saline
RT: Room temperature
