# Supplementary material for: Seven-year kinetics of RTS, S/AS01-induced anti-CSP antibodies in young Kenyan children
Source: Malar J. 2021 Dec 2;20:452. doi: 10.1186/s12936-021-03961-2 (PMC8641151; doi:10.1186/s12936-021-03961-2)
Supplement: Supplementary file 1 — Additional file 1. Decay patterns of the anti-CSP IgG and IgM antibodies. [file 12936_2021_3961_MOESM1_ESM.docx]

**Additional file 1**

## **Decay patterns of the anti-CSP IgG and IgM antibodies**


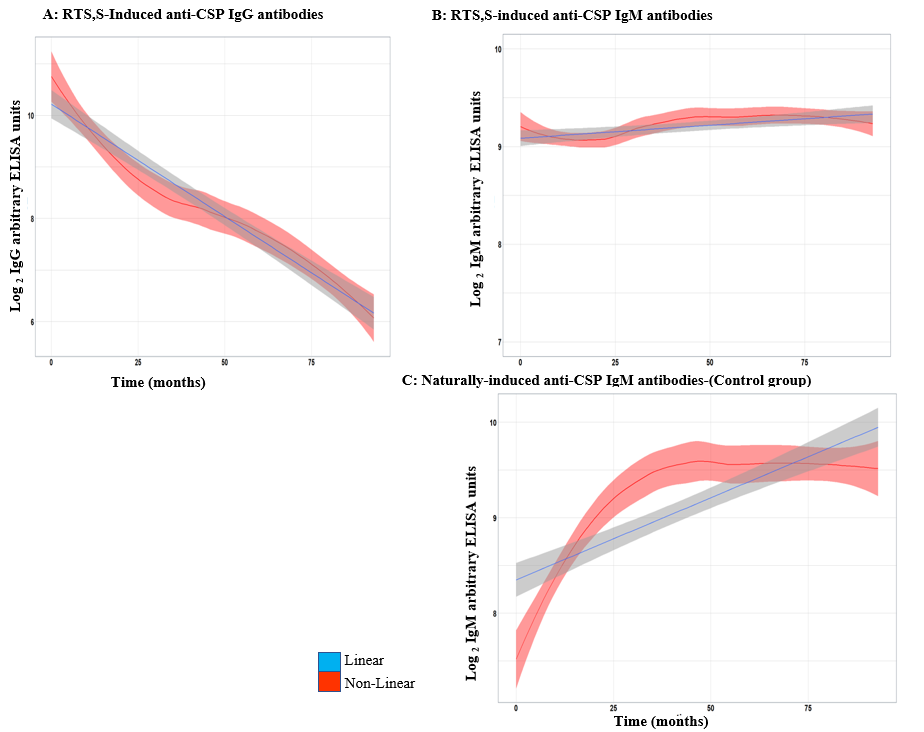


**Additional file 1**: Linear and non-linear modelling of RTS,S/AS01 induced anti-CSP (**a**) IgG, (**b**) IgM, and (**c**) natural exposure induced anti-CSP IgM Abs decay patterns. Linear model (blue) and non-linear model (red). The shades represent 95% confidence intervals.
