# Supplementary material for: Seven-year kinetics of RTS, S/AS01-induced anti-CSP antibodies in young Kenyan children
Source: Malar J. 2021 Dec 2;20:452. doi: 10.1186/s12936-021-03961-2 (PMC8641151; doi:10.1186/s12936-021-03961-2)
Supplement: Supplementary file 2 — Additional file 2. Kinetics of anti-tetanus IgG responses for the RTS,S/AS01, and control groups. [file 12936_2021_3961_MOESM2_ESM.docx]

## **Additional file 2**

## **Kinetics of anti-tetanus IgG responses for the RTS,S/AS01, and control groups**


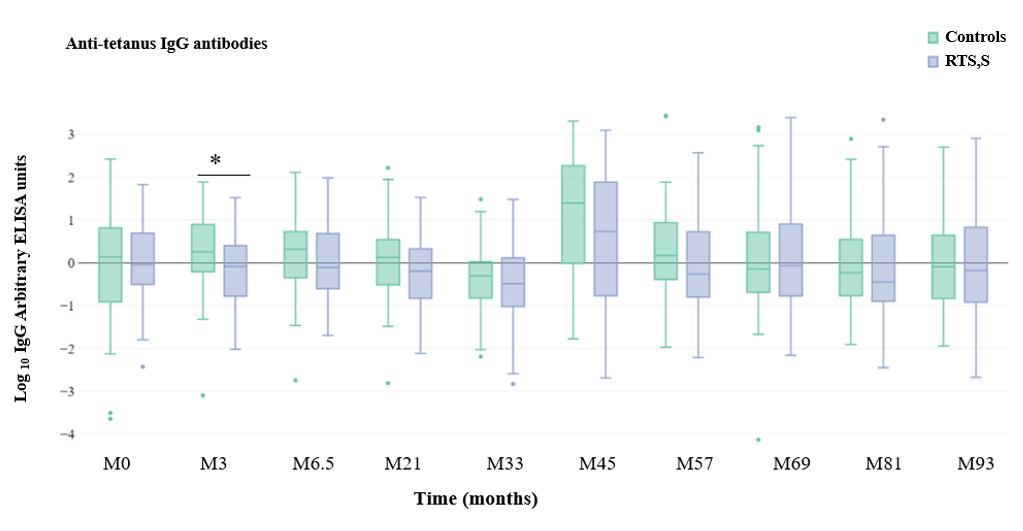


**Additional file 2**: Boxplots with medians Log_10_ concentrations of anti-tetanus IgG Abs. Whereby the top line represents 75^th^ percentile, the centerline represents median, and the bottom horizontal line shows the 25th percentile. The upper and lower whiskers represent the highest and lowest values within 1.5 interquartile ranges. The outlier’s AEU values are presented as dots. The statistical significance between the groups for each time point was determined using the Mann-Whitney U test, (M3 median of the control group=1.283, median of RTS,S/AS01 group=0.8900, Significance codes *P = 0.0215). Green represents the controls, n=49, blue represents RTS,S/AS01, n=50).
